# PCB118 Is Associated with Impaired Decidualization and Angiogenesis Through miR-542-3p–Mediated Regulation of ILK Signaling

**DOI:** 10.3390/ijms27093771

**Published:** 2026-04-23

**Authors:** Xinlan Qu, Yifan Sun, Yujie Yue, Yuan Fang, Songwei Lv

**Affiliations:** 1 Department of Obstetrics and Gynecology, Zhongnan Hospital of Wuhan University, Wuhan 430071, China; qxl_hao@126.com (X.Q.); yfsun@whu.edu.cn (Y.S.); 2 School of Pharmacy, Changzhou University, Changzhou 213164, China; s23091055043@smail.cczu.edu.cn; 3 Department of Thyroid and Mammary Gland Surgery, Zhongnan Hospital of Wuhan University, Wuhan 430071, China

**Keywords:** PCB118, epigenetic dysregulation, decidualization, angiogenesis

## Abstract

2,3′,4,4′,5-Pentachlorobiphenyl (PCB118) is a persistent environmental pollutant associated with adverse female reproductive outcomes; however, its effects on uterine function and epigenetic regulation remain incompletely understood. This study investigated whether PCB118 disrupts uterine decidualization and angiogenesis through miRNA-mediated regulatory pathways. Human endometrial stromal cells (HESCs) and human umbilical vein endothelial cells (HUVECs) were exposed to an environmentally relevant, non-cytotoxic concentration of PCB118. Decidualization and angiogenesis were evaluated in vitro, and underlying mechanisms were investigated using molecular and miRNA-based approaches. In vivo validation of miR-542-3p expression was performed in pregnant mice following PCB118 exposure. PCB118 exposure was associated with reduced expression of decidualization markers, including prolactin (PRL) and insulin-like growth factor-binding protein 1 (IGFBP-1), as well as impaired angiogenic capacity in HUVECs. PCB118 treatment was accompanied by increased miR-542-3p expression, which was associated with decreased integrin-linked kinase (ILK) levels and changes in transforming growth factor beta 1 (TGF-β1) and total Smad2 protein abundance. ILK overexpression partially restored decidualization and angiogenesis-related phenotypes, supporting a functional involvement of ILK in these processes. Consistently, elevated miR-542-3p expression was observed in murine endometrial tissues following PCB118 exposure, suggesting physiological relevance in vivo. PCB118 exposure is associated with impaired decidualization and angiogenesis, potentially involving dysregulation of the miR-542-3p/ILK signaling axis, suggesting a potential role for epigenetic modulation in PCB118-associated reproductive dysfunction.

## 1. Introduction

Decidualization is a critical adaptive process in mammalian pregnancy, characterized by the differentiation of HESCs, extracellular matrix remodeling, and coordinated angiogenesis [1]. This transformation establishes a receptive microenvironment that is essential for embryo implantation and placental development. Successful decidualization depends not only on finely regulated intracellular signaling but also on functional vascularization to supply oxygen and nutrients to the developing embryo [2]. Nevertheless, this finely tuned process is highly susceptible to disruption by environmental endocrine disruptors (EDCs), including polychlorinated biphenyls (PCBs), which can impair reproductive endocrine homeostasis, and the latter has recently been confirmed to have disrupting effects and the underlying mechanism on decidualization, a functional change in the endometrium preparing for pregnancy [3].

PCBs and their congeners, such as PCB118, are known to exert reproductive toxicity. Epidemiological and animal studies link PCB exposure to endometrial dysfunction, implantation failure, and miscarriage [4,5]. For instance, Richelle et al. [6] correlated serum PCB levels with reduced fertility in women, while Cai JL et al. [7] demonstrated that PCBs interfere with uterine receptivity and epithelial–mesenchymal transition. However, there remains a critical gap in the exploration of its toxic mechanisms. Specifically, systematic studies on how PCB118 exposure impairs decidualization and placental angiogenesis through post-transcriptional regulatory mechanisms are still lacking; furthermore, the role of non-coding RNA-mediated gene expression regulation in this process represents a core scientific question that urgently awaits clarification.

MicroRNAs (miRNAs) have emerged as key regulators in reproductive toxicology. By binding to target mRNA 3′ untranslated regions (3′-UTRs), miRNAs modulate gene expression involved in cell differentiation, proliferation, and vascularization [8]. Among these, miR-542-3p has been implicated in cancer, metabolic disorders, and notably reproductive system dysfunction [9,10,11]. Zhang et al. [12] further revealed that miR-542-3p can disrupt granulosa cell function via the insulin-like growth factor pathway. Emerging evidence indicates that microRNAs like miR-542-3p in placental mammals coordinate key pathways in endometrial epithelia, highlighting the importance of miRNA-mediated regulation in maintaining decidualization [13]. Notably, loss of miR-542-3p enhances IGFBP-1 expression in decidualizing human endometrial stromal cells [14], suggesting that miR-542-3p directly regulates decidualization-associated genes. Despite these advances, the role of miR-542-3p in decidualization and its dysregulation by environmental pollutants remains unexplored.

ILK, previously identified as a potential target of miR-542-3p in cancer cells [15], serves as a central node in cell adhesion, migration, and signal transduction [16,17,18]. Within the reproductive system, ILK is critical for embryo attachment and stromal decidualization [19,20]. Importantly, our study confirms that ILK is also a downstream target of miR-542-3p in decidualizing endometrial stromal cells [19], demonstrating that miR-542-3p negatively regulates decidualization through ILK signaling. The TGF-β1/Smad signaling pathway, a downstream regulatory cascade, is essential for endometrial remodeling and stromal cell differentiation [21,22]. Despite these advances, it remains unclear whether the miR-542-3p/ILK axis represents the primary mechanism through which PCB118 induces reproductive toxicity, highlighting the need for further studies to elucidate how PCB118-mediated miRNA dysregulation affects endometrial decidualization and overall reproductive function.

Based on this background, we hypothesize that PCB118 may upregulate miR-542-3p, leading to the suppression of ILK, disruption of TGF-β1/Smad signaling, and subsequent downregulation of angiogenic factors such as vascular endothelial growth factor (VEGF) and matrix metalloproteinase 9 (MMP9), thereby impairing the decidual microenvironment. This study employs integrated in vivo and in vitro approaches to systematically investigate the involvement of the miR-542-3p/ILK axis in PCB118-induced defects in decidualization and angiogenesis. By addressing this mechanistic gap, our work aims to provide novel molecular insights into how environmental pollutants compromise female reproductive health, supporting future biomarker development and targeted intervention strategies.

## 2. Results

### 2.1. Effects of PCB118 on Cell Viability, Apoptosis, and Invasion

**Figure 1:**
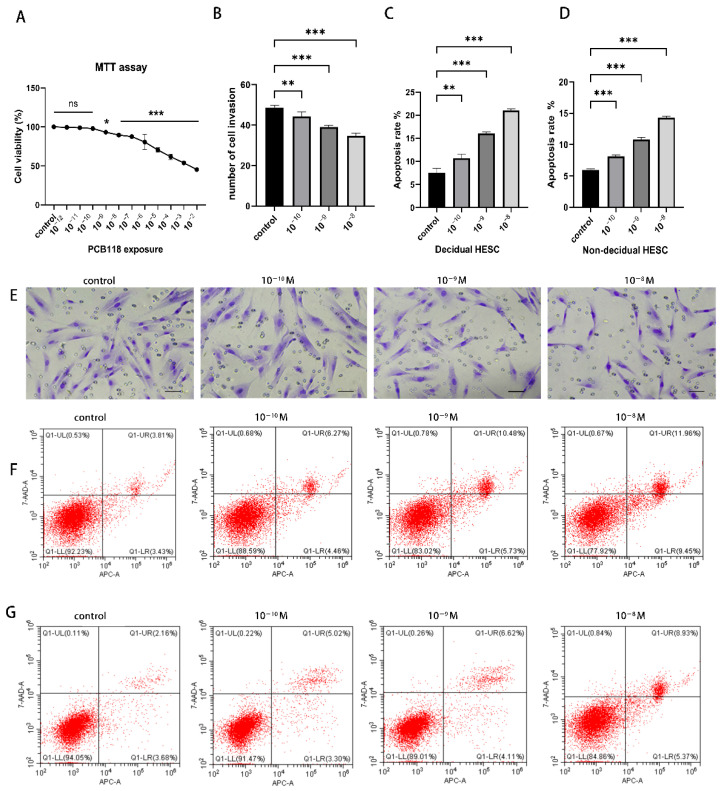
Effects of PCB118 exposure on the viability, apoptosis, and invasion of HESCs. (**A**) Cell viability of decidualized HESCs treated with increasing concentrations of PCB118 ranging from 10^−12^ to 10^−2^ M was assessed by MTT assay to determine a non-cytotoxic concentration for subsequent experiments. (**B**,**E**) Assessment of cell invasion capabilities in decidualized HESCs only. (**B**) Quantitative analysis of the relative number of invaded cells. (**E**) Representative images of the invasion assay (200× magnification), Scale bar = 20 μm. Data are presented as mean ± SD (*n* = 3). (**C**,**D**) Quantitative analysis of the apoptosis rate in decidualized (**C**) and non-decidualized (**D**) HESCs using flow cytometry analysis treated with non-cytotoxic concentrations (10^−10^ to 10^−8^ M) of PCB118. (**F**,**G**) Representative flow cytometry scatter plots of apoptosis in decidualized (**F**) and non-decidualized (**G**) HESCs. Red indicates the cell population. In the untreated group, most red cells are in the lower left quadrant (APC^−^/7-AAD^−^, live cells). Upon treatment with the substance, some red cells shift to the upper right quadrant (APC^+^/7-AAD^+^, late apoptotic/dead cells). Immortalized HESCs were induced to decidualize for 48 h, after which PCB118 was added and cells were further cultured for 24 h. Statistical significance among multiple groups was determined using one-way ANOVA followed by Tukey’s post hoc test. * *p* < 0.05, ** *p* < 0.01, *** *p* < 0.001 versus control group; ns, not significant; horizontal lines indicate comparisons with control.

HESCs were exposed to various concentrations of PCB118 (Figure 1A). Cell viability was significantly reduced in cells treated with 10^−9^–10^−3^ M PCB118, reaching 0.55–0.93-fold (*p* < 0.01). In contrast, no significant changes in viability were observed at lower concentrations (10^−12^–10^−10^ M). To minimize the influence of cytotoxicity, concentrations with minimal impact on viability (10^−10^ M to 10^−8^ M) were selected for subsequent apoptosis and invasion assays.

Flow cytometric analysis revealed a concentration-dependent increase in apoptosis in both decidualized and non-decidualized HESCs, with apoptotic rates elevated by approximately 1.3- to 2.8-fold compared with the control (Figure 1C,D). In addition, cell invasion was significantly inhibited by 9.0% to 28.8% at concentrations ranging from 10^−10^ M to 10^−8^ M (*p* < 0.05; Figure 1C,E). Notably, PCB118-induced apoptosis was more pronounced in decidualized HESCs than in non-decidualized cells.

At the selected concentration of 10^−9^ M, cell viability, as assessed by MTT assay, remained at approximately 93% of control (a 7% reduction), while the suppression of functional endpoints—including apoptosis and invasion—reached magnitudes substantially exceeding this minimal cytotoxicity (as detailed in Section 2.2). This discrepancy suggests that the observed functional alterations cannot be fully explained by reduced cell viability alone and are consistent with effects occurring under largely non-cytotoxic conditions.

### 2.2. PCB118 Exposure Induces Decidualization Impairment

**Figure 2:**
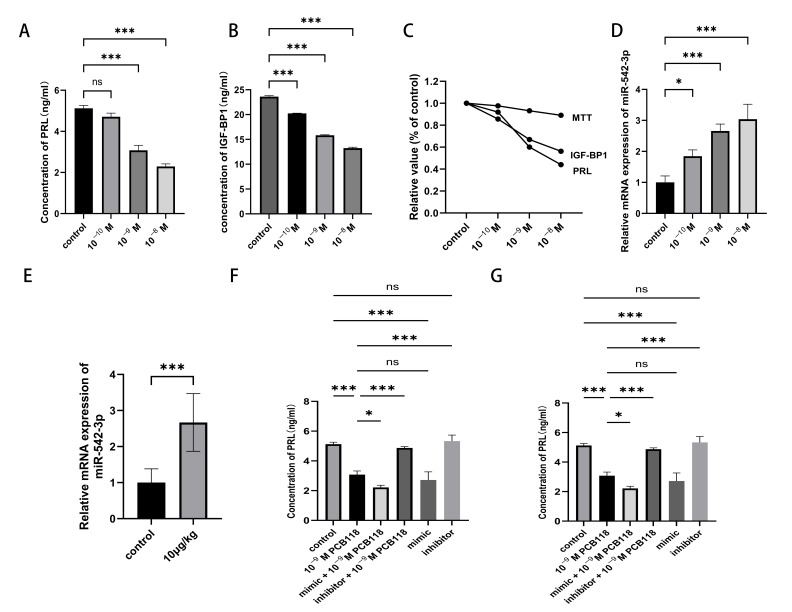
PCB118 exposure was associated with decreased expression of decidualization markers, including PRL and IGFBP1 in HESCs through upregulation of miR-542-3p. (**A**,**B**) HESCs were induced for decidualization using cAMP and MPA and treated with PCB118 (10^−10^ M to 10^−8^ M). The secretion levels of PRL (**A**) and IGFBP-1 (**B**) were quantified by ELISA. (**C**) Concentration-dependent effects of PCB118 on cell viability, decidualization markers (PRL, IGFBP-1) (**D**,**E**) Relative mRNA expression levels of miR-542-3p in 72 h decidualized HESCs (**D**) and decidual tissues at day 8 of pregnancy (**E**) following PCB118 exposure were determined by qRT-PCR. (**F**,**G**) Functional analysis of miR-542-3p in decidualization. HESCs were transfected with miR-542-3p mimics or inhibitors, or combined with PCB118, and PRL (**F**) and IGFBP-1 (**G**) secretion were assessed by ELISA. For experiments involving decidualized HESCs, cells were induced to decidualize for 48 h, followed by 48 h of transfection, with PCB118 added during the final 24 h of the transfection period (total decidualization duration: 96 h). For experiments without transfection, cells were induced to decidualize for 48 h, followed by 24 h of PCB118 treatment (total decidualization duration: 72 h), as indicated. Data are presented as mean ± SD. *n* = 3 for in vitro experiments and *n* = 7 for decidual tissue analyzes. Data are presented as mean ± SD. Statistical significance among multiple groups was determined using one-way ANOVA followed by Tukey’s post hoc test. Two groups were determined using an unpaired Student’s *t*-test. * *p* < 0.05, *** *p* < 0.001 versus control group; ns, not significant.

Decidualization was induced in HESCs using cAMP and MPA, and the concentration of decidualization markers prolactin (PRL) and insulin-like growth factor binding protein-1 (IGFBP-1) was evaluated. Exposure to 10^−10^ M PCB118 did not significantly affect PRL levels; however, 10^−9^ M and 10^−8^ M PCB118 significantly reduced PRL secretion to 60% and 40% of the control, respectively (*p* < 0.01; Figure 2A). In contrast, IGFBP-1 concentration was significantly suppressed across all PCB118-treated groups, ranging from 57% to 84% of the control levels (*p* < 0.01; Figure 2B). Based on these results and the minimal cytotoxicity observed at 10^−9^ M (cell viability: 93% of control, as shown in Section 2.1), this concentration was selected for further molecular analyses (Figure 2A).

### 2.3. miR-542-3p Is Associated with PCB118-Induced Decidualization Defects

The expression of miR-542-3p was significantly upregulated by approximately 1.8- to 3.0-fold in PCB118-treated decidual stromal cells compared with controls (*p* < 0.01; Figure 2C). This trend was consistent with observations in a mouse decidual model exposed to 10 μg/kg/d PCB118 on day 8 of pregnancy (*p* < 0.01; Figure 2D). Transfection with miR-542-3p mimics further reduced PRL and IGFBP-1 secretion to 0.71-fold and 0.68-fold of control levels, respectively, whereas inhibition of miR-542-3p restored their levels (*p* < 0.01; Figure 2E,F).

### 2.4. miR-542-3p Regulates ILK Expression Under PCB118 Exposure

**Figure 3:**
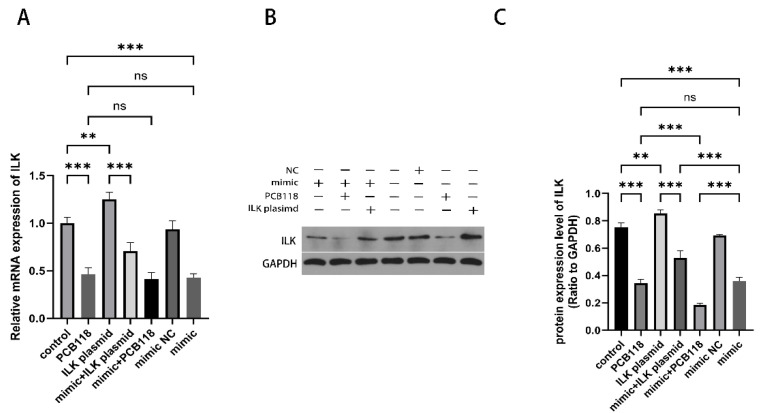
ILK serves as a downstream target of miR-542-3p in 10^−9^ M PCB118-induced decidualization impairment. HESCs were induced to decidualize for 48 h, followed by 48 h of transfection, with PCB118 added during the final 24 h of the transfection period. (**A**) Relative mRNA expression levels of ILK in HESCs were analyzed by qRT-PCR. Cells were treated with PCB118, transfected with miR-542-3p mimic, or co-transfected with an ILK overexpression plasmid (pcDNA3.1-ILK). (**B**,**C**) Western blot analysis of ILK protein expression under the same experimental conditions. (**B**) Representative Western blot images showing ILK protein bands. “+” and “−” indicate the presence or absence of the indicated exposures/treatments. (**C**) Quantitative analysis of ILK protein levels normalized to the loading control. Data are presented as mean ± SD (*n* = 3). Statistical significance among multiple groups was determined using one-way ANOVA followed by Tukey’s post hoc test. ** *p* < 0.01, *** *p* < 0.001 versus control group; ns, not significant.

ILK expression was examined at both the mRNA and protein levels under different treatments (Figure 3). PCB118 treatment significantly reduced ILK expression at both the mRNA and protein levels compared with the control group (*p* < 0.01). Transfection with miR-542-3p mimic alone also significantly reduced ILK expression at both mRNA and protein levels compared with the control group (*p* < 0.05), and there was no significant difference between the mimic alone and PCB118 treatment groups (*p* > 0.05). ILK plasmid transfection significantly increased ILK expression compared with the control group (*p* < 0.05). Co-transfection with miR-542-3p mimic significantly decreased ILK expression at the mRNA and protein levels compared with the ILK plasmid group (*p* <0.01), while no significant differences were observed in the mimic negative control group (*p* > 0.05). In addition, combined treatment with miR-542-3p mimic and PCB118 resulted in significantly lower ILK protein levels than either PCB118 treatment alone or mimic alone (*p* < 0.05; Figure 3B,C), while no significant difference was found between the two individual treatments (*p* > 0.05; Figure 3B,C). At the mRNA level, the combined treatment did not differ significantly from either treatment alone (*p* > 0.05), indicating that the synergistic effect on ILK expression was specific to the protein level.

### 2.5. Alterations in TGF-β1 and Total Smad2 Protein Levels Following Modulation of miR-542-3p and ILK Expression

**Figure 4:**
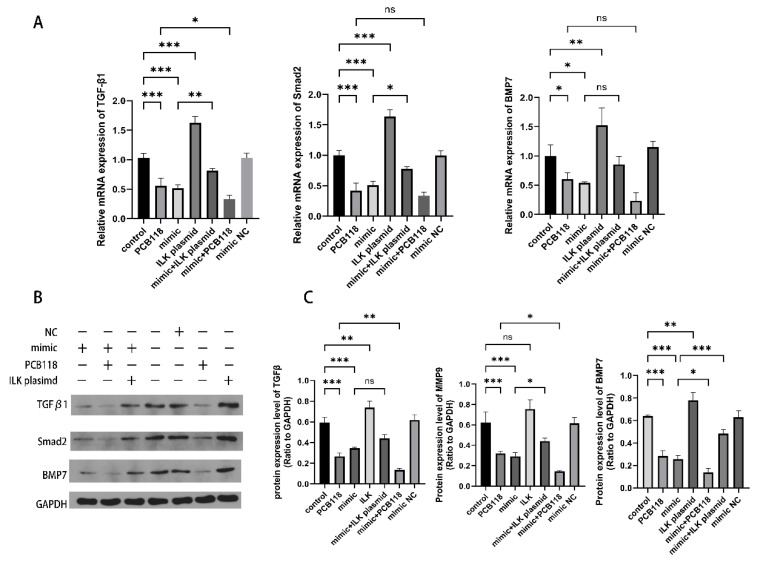
10^−9^ M PCB118 reduces TGF-β1 and total Smad2 protein levels via the miR-542-3p/ILK axis. HESCs were induced to decidualize for 48 h, followed by 48 h of transfection, with PCB118 added during the final 24 h of the transfection period. (**A**) Relative mRNA expression levels of TGF-β1, Smad2, and BMP7 in HESCs were quantified by qRT-PCR. Cells were treated with PCB118, transfected with a miR-542-3p mimic, or co-transfected with an ILK overexpression plasmid (pcDNA3.1-ILK) to evaluate the regulatory effects on gene expression. (**B**,**C**) Western blot analysis of TGF-β1, Smad2 and BMP7 protein expression. The figure displays representative protein bands and the corresponding quantitative statistical analysis. “+” and “−” indicate the presence or absence of the indicated exposures/treatments. Data are presented as mean ± SD (*n* = 3). Statistical significance among multiple groups was determined using one-way ANOVA followed by Tukey’s post hoc test. * *p* < 0.05, ** *p* < 0.01, *** *p* < 0.001 versus control group; ns, not significant.

PCB118 exposure and transfection with a miR-542-3p mimic significantly downregulated TGF-β1 and Smad2 protein expression by approximately 50% compared with the control group (*p* < 0.01; Figure 4A). Transfection with miR-542-3p inhibitor significantly increased TGF-β1 and Smad2 protein levels compared with the inhibitor negative control (NC) group (*p* < 0.05), while no significant difference was observed between the inhibitor NC and control groups (*p* > 0.05; Figure S1). Overexpression of ILK markedly increased the mRNA expression levels of TGF-β1 (*p* < 0.01; Figure 4A), whereas no significant change in Smad2 mRNA expression was observed (*p* > 0.05; Figure 4C). However, ILK overexpression in miR-542-3p mimic–treated cells led to an increase in Smad2 protein levels compared with the miR-542-3p mimic group (*p* < 0.05; Figure 4C), while TGF-β1 protein expression remained unchanged. In addition, combined treatment with PCB118 and the miR-542-3p mimic resulted in significantly lower TGF-β1 and Smad2 protein levels compared with PCB118 treatment alone (*p* < 0.05; Figure 4C).

Furthermore, both PCB118 exposure and miR-542-3p mimic transfection significantly downregulated BMP7 mRNA and protein expression. Notably, BMP7 protein levels were significantly elevated in the pcDNA3.1-ILK plus miR-542-3p mimic group compared with the miR-542-3p mimic group alone (*p* < 0.05; Figure 4).

### 2.6. Effects of PCB118, miR-542-3p, and ILK on Angiogenesis-Related Responses

**Figure 5:**
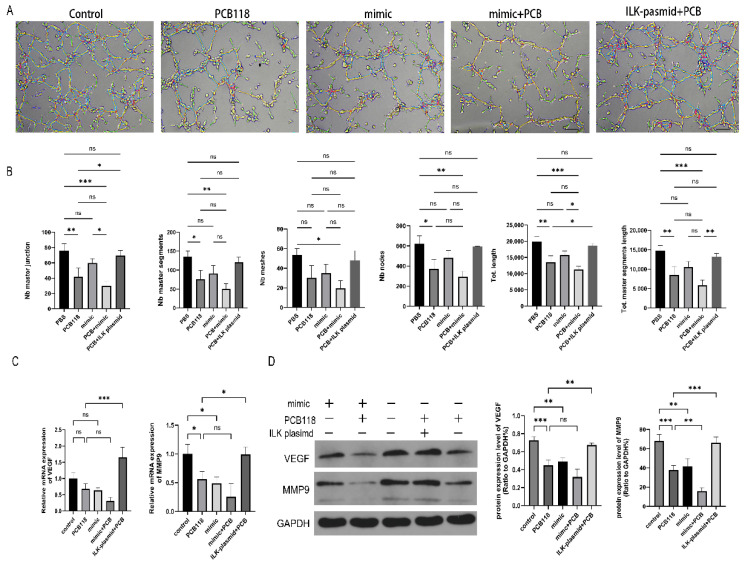
ILK overexpression partially restores 10^−9^ M PCB118-induced angiogenic dysfunction in HUVECs. HESCs were induced to decidualize for 48 h, followed by 48 h of transfection, with PCB118 added during the final 24 h of the transfection period. Conditioned media were collected from HESCs subjected to the indicated treatments (PCB118, miR-542-3p mimic, and/or pcDNA3.1-ILK) and used to incubate HUVECs for angiogenic capacity evaluation. (**A**) Representative images of the tube formation assay analyzed by ImageJ Angiogenesis Analyzer (version: 1.54g, 100× magnification), Scale bar = 20 μm. Color coding: red lines = master segments (main vascular trunks), yellow lines = slave segments (secondary branches), green dots = junctions (vascular connection points), purple areas = meshes (vascular loops), blue dots = end points (vascular free ends). (**B**) Quantitative analysis of tube formation parameters, including the number (Nb) of master junctions, master segments, meshes, and nodes, as well as the total length and total master segments length. (**C**) Relative mRNA expression levels of the angiogenesis-related factors VEGF and MMP9 in HUVECs assessed by qRT-PCR. (**D**) Western blot analysis of VEGF and MMP9 protein expression. The figure displays representative protein bands and the corresponding quantitative statistical analysis. “+” and “−” indicate the presence or absence of the indicated exposures/treatments. Data are presented as mean ± SD (*n* = 3). Statistical significance among multiple groups was determined using one-way ANOVA followed by Tukey’s post hoc test. * *p* < 0.05, ** *p* < 0.01, *** *p* < 0.001 versus control group; ns, not significant.

PCB118 exposure significantly impaired tube formation in HUVECs compared with the control group (*p* < 0.01; Figure 5A). Consistently, tube length, the number of master junctions, and the number of nodes were markedly reduced in PCB118-exposed HUVECs (*p* < 0.01; Figure 5B). Moreover, the protein expression levels of angiogenesis-related factors, including VEGF and MMP9, were significantly decreased following PCB118 exposure (*p* < 0.01; Figure 5D). Transfection with a miR-542-3p mimic produced similar inhibitory effects on tube formation and angiogenic protein expression. Co-treatment with PCB118 and the miR-542-3p mimic did not further alter tube formation capacity compared with PCB118 treatment alone (*p* > 0.05; Figure 5B); however, MMP9 protein expression was further reduced (*p* < 0.01; Figure 5D). Notably, overexpression of ILK significantly increased tube length and markedly elevated VEGF and MMP9 protein expression levels in PCB118-treated HUVECs (*p* < 0.05; Figure 5B–D).

## 3. Discussion

This study investigates the effects of PCB118 on endometrial stromal function using a combination of in vitro and in vivo approaches, including cell culture, flow cytometry, and molecular biology analyses. By integrating cellular functional assays with mechanistic analyzes and animal exposure data, we provide evidence that PCB118 exposure is associated with impaired decidualization and angiogenic capacity, potentially mediated by dysregulation of the miR-542-3p/ILK signaling axis.

### 3.1. PCB118 Exposure and Endometrial Stromal Cell Dysfunction

Epidemiological studies have reported associations between PCB exposure and adverse female reproductive outcomes, including prolonged time to pregnancy and reduced fecundity [23,24]. Within this context, our in vitro findings provide experimental observations that are consistent with a potential role of PCB118 in disrupting endometrial function. Rather than establishing a direct mechanistic pathway, the present data indicate that PCB118 exposure is associated with functional impairment of stromal cells under the tested conditions.

PCB118 induced dose-dependent cytotoxicity in HESCs at high concentrations, while environmentally relevant non-cytotoxic concentrations were sufficient to impair stromal cell invasion, increase apoptosis, and suppress the expression of classical decidualization markers PRL and IGFBP-1. It should be noted that decidualization in the present study was assessed primarily based on PRL and IGFBP1 expression. Although these are well-established markers of stromal decidualization, they represent only selected aspects of the decidual phenotype and do not fully capture the complexity of the decidualization process. Therefore, the observed changes should be interpreted as alterations in decidualization-associated marker expression rather than definitive evidence of global impairment of decidualization. Morphological assessment was not performed because the relatively early stage of decidualization induction did not allow clear morphological differentiation.

Importantly, these functional disturbances were observed under conditions in which no substantial reduction in cell viability was detected, as assessed by MTT assay, indicating minimal cytotoxicity. Under these conditions, PCB118 exposure was associated with changes in stromal cell function that cannot be fully explained by reduced viability alone. Although a minor contribution from mild apoptosis cannot be completely excluded, the overall pattern of evidence is consistent with effects occurring predominantly under non-cytotoxic conditions. These findings are based on experimental conditions in which cell viability was largely preserved, supporting interpretation within a predominantly non-cytotoxic context. This observation is consistent with the concept that endocrine-disrupting chemicals can exert biologically significant effects at low doses, which may precede or occur without obvious histopathological damage.

To further clarify whether the decidualization state influences cellular susceptibility to PCB118, apoptosis assays were performed in both non-decidualized and decidualized HESCs. PCB118 exposure was associated with increased apoptosis in both cell types; however, decidualized cells exhibited significantly higher apoptotic rates than non-decidualized cells at all tested concentrations. These results suggest that the decidualization state may be associated with increased cell sensitivity to PCB118-induced cellular stress. 

Because decidualized stromal cells acquire specialized functions such as enhanced invasiveness and endocrine activity, which are essential for embryo implantation and placental development, subsequent functional assessments (including viability and invasion assays) were conducted primarily in this physiologically relevant state. This approach reflects the biological reality that non-decidualized stromal cells generally do not exhibit invasive behavior in vivo, making the decidualized model more appropriate for evaluating implantation-related toxicity.

The heightened sensitivity of decidualized cells to PCB118-induced apoptosis further highlights the potential vulnerability of the implantation window to environmental toxicants. The consistency between our experimental observations and epidemiological evidence strengthens the biological plausibility that PCB118 exposure may contribute to implantation-related reproductive dysfunction, although causal relationships cannot be established based on the present data.

### 3.2. miR-542-3p Potentially Involved in PCB118-Associated Alterations in Decidualization-Related Processes

miRNAs have emerged as critical mediators linking environmental exposures to altered cellular phenotypes. Previous studies have demonstrated that PCB exposure is associated with changes in circulating and tissue-specific miRNA profiles [25,26,27]; however, the role of miR-542-3p in endometrial biology has remained poorly defined.

miR-542-3p has been extensively studied in various diseases such as cancer [15,28,29], neurological diseases [30], and cardiovascular disorders [31], where it regulates cell proliferation, invasion, and extracellular matrix remodeling. In endometrial-related contexts, emerging evidence has suggested that miR-542-3p may participate in the regulation of genes critical for stromal function. For example, studies in embryonic stem cell–derived endometrial models and endometriosis have indicated that miR-542-3p can modulate the expression of PRL, IGFBP1, and WNT4, thereby influencing cell migration and invasion in ectopic or non-physiological environments [14].

Although early pregnancy shares certain biological features with tumor progression, including dynamic cellular differentiation and migration, the role of miR-542-3p in physiological decidualization, particularly under environmental pollutant exposure, remains poorly defined. In the present study, PCB118 exposure was associated with upregulation of miR-542-3p in HESCs and in decidual tissues from exposed pregnant mice. Functional modulation experiments further suggested that inhibition of miR-542-3p partially alleviated the suppression of PRL and IGFBP1 observed under control conditions, whereas miR-542-3p overexpression exacerbated these effects.

### 3.3. Involvement of the miR-542-3p/ILK Axis in Stromal Signaling Regulation

Building on our previous identification of ILK as a direct downstream target of miR-542-3p in decidualizing human endometrial stromal cells (HESCs) [19], the present study extends previous findings by showing that PCB118 exposure is associated with coordinated changes in miR-542-3p expression and ILK levels, consistent with perturbation of the miR-542-3p/ILK axis. Notably, restoration of ILK expression partially rescued PCB118-induced decidual defects, supporting the functional relevance of ILK downregulation in this context rather than establishing a direct causal role. 

At the signaling-related expression level, our data further suggest that reduced ILK expression was associated with decreased TGF-β1 expression and total Smad2 protein abundance, both of which have been implicated in stromal decidualization [32,33]. Functional modulation of miR-542-3p further supported this association, as overexpression of miR-542-3p using a mimic enhanced the suppression of downstream signaling components, whereas inhibition of miR-542-3p significantly increased TGF-β1 and Smad2 protein expression compared with the inhibitor negative control. Together, these experiments are consistent with a role of miR-542-3p in the regulation of ILK-associated signaling in decidual stromal cells.

It is noteworthy that the regulatory effects of the miR-542-3p/ILK axis were not uniformly reflected at the transcriptional, translational, and functional levels. For example, ILK overexpression significantly increased TGF-β1 mRNA expression, whereas Smad2 mRNA levels remained unchanged, despite an increase in Smad2 protein abundance under miR-542-3p mimic conditions. This discrepancy suggests that ILK may modulate Smad2 primarily through post-transcriptional or post-translational mechanisms, such as protein stabilization or signaling complex assembly, rather than direct transcriptional regulation. Such multilayered regulation is consistent with the known scaffolding and signaling functions of ILK and highlights the complexity of stromal signaling responses under environmental stress.

It should be noted that the miR-542-3p inhibitor experiments were conducted in the absence of PCB118 exposure; therefore, combined treatment with PCB118 was not evaluated in the present study. Future investigations incorporating both PCB118 exposure and miR-542-3p inhibition will be necessary to further clarify the interactive regulatory mechanisms underlying toxicant-induced decidualization impairment.

Although phosphorylation of Smad2 was not directly evaluated in this study, the observed alterations are compatible with a model in which disruption of ILK-dependent signaling contributes to PCB118-induced decidual dysfunction. Moreover, given the multifaceted signaling role of ILK, including its involvement in PI3K/Akt signaling [34], additional downstream pathways may also participate in the observed phenotypes and represent important directions for future investigation.

### 3.4. Disruption of Angiogenic Processes and Endothelial Model Considerations

Successful decidualization is tightly coupled with coordinated angiogenesis to support embryo implantation and placental development. Using HUVEC models as a well-established in vitro endothelial model [34,35], we found that PCB118 exposure impaired tube formation and reduced the expression of angiogenic factors VEGF and MMP9. These effects were phenocopied by miR-542-3p overexpression and partially rescued by ILK restoration, suggesting that the miR-542-3p/ILK axis may also be involved in the regulation of angiogenic responses under PCB118 exposure. However, given the complexity of angiogenic signaling, these observations should be interpreted as indicative of functional association rather than direct pathway mediation. Interestingly, although combined treatment with PCB118 and the miR-542-3p mimic further reduced MMP9 expression, tube formation capacity was not further impaired compared with PCB118 treatment alone. This suggests a threshold effect in angiogenic regulation, whereby additional molecular suppression does not result in proportional functional changes, reflecting the integrated and multicomponent nature of angiogenic signaling.

Taken together, our findings support a working model in which PCB118 exposure is associated with upregulation of miR-542-3p, leading to suppression of ILK and subsequent impairment of decidualization-related signaling and angiogenic factor expression. Rather than defining a definitive pathway, this model provides a mechanistic framework linking environmental PCB exposure to coordinated stromal and endothelial dysfunction at the maternal–fetal interface.

### 3.5. Study Limitations

Several limitations of this study should be acknowledged. First, the use of immortalized HESCs may not fully reflect the signaling dynamics, epigenetic landscape, or miRNA responsiveness of primary human endometrial stromal cells. Similarly, although HUVECs offer standardized and reproducible assays for quantifying endothelial responses, which makes them highly suitable for mechanistic studies of pollutant-induced angiogenic disruption [35,36]. However, as large-vessel endothelial cells, HUVECs do not fully recapitulate the specialized uterine microvascular endothelium involved in implantation-site angiogenesis. Therefore, extrapolation of in vitro findings to the in vivo uterine microenvironment should be made with caution, and future studies using primary decidual endothelial cells or endometrial organoid co-culture models are warranted to validate these results. Second, the functional assays used in this study have certain constraints. For example, uncoated transwell migration assays were not performed, making it difficult to distinguish between alterations in cell motility and extracellular matrix remodeling. As a result, the observed changes in invasion likely reflect a combination of these processes. Third, methodological limitation concerns RT-qPCR normalisation. Although geNorm recommends geometric mean normalisation of multiple stable reference genes, we used a single validated gene (GAPDH, U6) across all samples because other candidates were measured only in a subset; GAPDH was stable, but multiple genes would improve robustness. Fourth, the in vivo exposure regimen does not fully replicate human environmental conditions. The PCB118 dose used in the present in vivo study (10 µg/kg/day) was higher than the estimated typical human environmental exposure (1.8–3.6 µg/kg/day based on reported concentrations of 9.05–18.2 ng/g lipid) [37,38]. When adjusted for body surface area, the human-equivalent dose for mice is approximately 20–40 µg/kg/day. Nevertheless, this regimen does not fully replicate chronic low-dose exposure in humans. While we observed miR-542-3p upregulation in the decidual tissues of PCB118-treated mice, PRL and IGFBP-1 expression in these tissues was not measured. Therefore, direct confirmation of decidualization suppression in vivo remains to be investigated in future studies. Fifth, the mechanistic scope of the study is limited. The analyses focused on a single miRNA and did not comprehensively assess downstream signaling intermediates such as p-Smad2 or p-AKT, limiting pathway resolution. In addition, although previous studies have demonstrated the direct targeting of ILK by miR-542-3p, the present study did not perform a luciferase reporter assay in the presence of PCB118, which would provide more direct evidence regarding modulation of this interaction. Sixth, species differences should be considered when interpreting the in vivo findings. Although murine models are commonly used to investigate reproductive toxicity of environmental pollutants, differences in endometrial physiology between mice and humans may limit direct translational relevance. Due to experimental constraints, some in vivo validation experiments could not be further expanded. Nevertheless, the consistency between in vitro cellular results and observations in the mouse decidual model supports the biological relevance of our findings. Importantly, although functional modulation experiments were performed, the present study does not establish a direct causal relationship between PCB118 exposure and miR-542-3p/ILK-mediated signaling. Instead, the findings are consistent with involvement of this regulatory axis, and further studies using more targeted mechanistic approaches will be required to clarify causal relationships.

### 3.6. Future Research Directions

Future studies using primary human endometrial stromal cells and uterine microvascular endothelial cells, as well as chronic low-dose PCB118 exposure models that better reflect human environmental conditions, will be important to validate the translational relevance of our findings. We acknowledge that the current findings represent preliminary evidence exploring the potential mechanistic role of miR-542-3p in PCB118-induced endothelial dysfunction.

PCB118 is a dioxin-like polychlorinated biphenyl capable of activating the AhR signaling pathway and inducing oxidative stress. Previous studies have shown that environmental toxicants can modulate microRNA expression through AhR-dependent transcriptional regulation or oxidative stress-associated signaling pathways. Although the precise mechanism by which PCB118 induces miR-542-3p expression was not investigated in the present study, it is plausible that PCB118 regulates miR-542-3p through these pathways. Specifically, future studies employing promoter-reporter assays and luciferase assays conducted in the presence of PCB118 will be necessary to determine whether PCB118 directly modulates miR-542-3p transcription through AhR-dependent or oxidative stress-mediated pathways. In addition, since transfection was performed after decidualization induction, our findings mainly reflect the role of miR-542-3p in the maintenance and progression of decidualization rather than its initiation. Whether miR-542-3p also participates in the initiation phase of decidualization remains to be determined.

Finally, expanding future analyses to include multi-miRNA regulatory networks, comprehensive phospho-signaling profiling, and clinical endometrial samples from PCB-exposed women will be important for further elucidating the molecular basis of PCB-associated reproductive dysfunction.

## 4. Materials and Methods

### 4.1. Cell Culture and Decidualization

The immortalized HESC line was cultured as described in our previous studies [19]. HESCs were maintained in DMEM/F12 medium (Gibco, Waltham, MA, USA) supplemented with 10% charcoal-stripped fetal bovine serum (CS-FBS; Biological Industries, Beit Haemek, Israel) at 37 °C in a humidified atmosphere of 5% CO_2_. Cells were synchronized at the G0/G1 phase by overnight serum starvation. Decidualization was induced in vitro using differentiation medium containing DMEM/F12 with 2% CS-FBS, 10 nM of estradiol (E2; Sigma-Aldrich, St. Louis, MO, USA; E8875), 1 µM of medroxyprogesterone 17-acetate (MPA; Sigma-Aldrich, Steinheim, Germany), and 0.5 mM of dibutyryl cAMP (dbcAMP; Sigma-Aldrich, Steinheim, Germany). After 48 h of decidualization induction, cells were either harvested or subjected to further treatments as described below. For experiments involving transfection and PCB118 exposure, the differentiation medium was maintained throughout the entire culture period to preserve the decidualized phenotype. The total duration of decidualization prior to sample collection was 96 h. For experiments not involving transfection, the total decidualization time was 72 h.

PCB118 was purchased from AccuStandard Inc. (New Haven, CT, USA; 31508-00-6). The stock solution of PCB118 was prepared by dissolving the powder in anhydrous dimethyl sulfoxide (DMSO, Sigma-Aldrich, St. Louis, MO, USA; D8418) to a concentration of 10^−2^ M, and then serially diluted with serum-free DMEM/F12 medium to obtain working concentrations ranging from 10^−12^ M to 10^−2^ M. For treatment, HESCs were independently exposed to the above gradient concentrations of PCB118, with 0.1% DMSO-containing medium as the vehicle control (to eliminate potential solvent effects), for 24 h. The culture medium was refreshed every 24 h during the treatment period.

### 4.2. Plasmid Construction

The coding sequence (CDS) of ILK was cloned into the pcDNA3.1(+) vector (Invitrogen, Thermo Fisher Scientific, Waltham, MA, USA; V79020) to generate pcDNA3.1-ILK. All constructs were verified by DNA sequencing.

### 4.3. Oligonucleotide and Plasmid Transfection

HESCs were induced to decidualize for 48 h as described in Section 4.1. After induction, cells were transfected with miR-542-3p mimics or inhibitors (MedChemExpress, Monmouth Junction, NJ, USA; HY-R01624/ HY-RI01624), corresponding negative controls, or pcDNA3.1-ILK using Lipofectamine 2000 (Invitrogen, Thermo Fisher Scientific, Waltham, MA, USA), following the manufacturer’s instructions. In brief, 5 µL of plasmid DNA or 20 nM of miRNA mimic/inhibitor was diluted in 100 µL of serum-free Opti-MEM (Gibco; Waltham, MA, USA), mixed with 4 µL of Lipofectamine 2000 diluted in 100 µL Opti-MEM, incubated at room temperature for 5 min, and added to the cells. The transfection complexes were incubated with cells for 6 h, after which the medium was replaced with fresh complete medium to reduce potential cytotoxicity. Cells were then cultured for an additional 42 h to achieve a total transfection time of 48 h at 37 °C and 5% CO_2_ before subsequent experiments.

For experiments involving PCB118 (10^−9^ M), the compound was administered during the last 24 h of the 48 h transfection period, commencing 24 h after the medium was replaced following transfection. All transfection experiments were performed in triplicate. Control groups were treated with vehicle (0.1% DMSO) and/or the corresponding negative control oligonucleotides or empty vector, as applicable.

### 4.4. Enzyme-Linked Immunosorbent Assay (ELISA) for Decidualization Markers

The concentrations of prolactin (PRL) and insulin-like growth factor-binding protein 1 (IGFBP-1) in HESC culture supernatants were measured using commercial ELISA kits (Elabscience Biotechnology, Wuhan, China; PRL E-EL-H0047, IGFBP-1 E-EL-H1089). Cells were assigned to the following groups: Control group, PCB118 (10^−9^ M, 24 h) alone, miR-542-3p mimic plus PCB118, miR-542-3p inhibitor plus PCB118. All groups were treated according to the standard protocol described in Section 4.1 and Section 4.3: cells were decidualized for 48 h, followed by 48 h of transfection, with PCB118 (10^−9^ M) added during the final 24 h. Supernatants were collected at the end of the 96 h total decidualization period. The absorbance of each well was detected at a wavelength of 450 nm with a reference wavelength of 540 nm using a Multiskan FC Microplate Photometer (Thermo Fisher Scientific, Waltham, MA, USA) according to the manufacturer’s instructions. All measurements were performed in triplicate and normalized to total cellular protein content.

### 4.5. Cell Viability Assay

Cell viability was assessed using the MTT assay in decidualized HESCs to determine a non-cytotoxic concentration of PCB118 for subsequent experiments. HESCs were first induced to undergo decidualization for 48 h as described in Section 4.1, followed by exposure to increasing concentrations of PCB118 (10^−12^ M to 10^−2^ M) for 24 h. Following treatment, MTT solution (5 mg/mL, Sigma-Aldrich, St. Louis, MO, USA; M2128) was added to each well and incubated for 4 h at 37 °C. The supernatant was then carefully removed, and the resulting formazan crystals were dissolved in dimethyl sulfoxide (DMSO). Absorbance was measured at 570 nm using a Multiskan FC Microplate Photometer (Thermo Fisher Scientific, Waltham, MA, USA). Based on these results, a non-cytotoxic concentration of PCB118 (10^−9^ M) was selected for all subsequent experiments. All experiments were performed in triplicate.

### 4.6. Cell Invasion Assay

Cell invasion was evaluated in decidualized HESCs using Transwell chambers (BD Biosciences, San Jose, CA, USA; 353097) pre-coated with Matrigel (Corning, NY, USA; 356234), which assesses the ability of cells to migrate through an extracellular matrix barrier. To ensure consistency, the same batch and dilution of Matrigel were used for all experimental groups. The Matrigel layer was allowed to polymerize under identical conditions before cell seeding. After serum starvation for 24 h, 2 × 10^5^ cells suspended in 200 µL serum-free medium were seeded into the upper chamber, while the lower chamber was filled with medium containing 10% fetal bovine serum as a chemoattractant. Cells were incubated for the indicated time at 37 °C in a humidified atmosphere containing 5% CO_2_. After incubation, non-invading cells on the upper surface of the membrane were removed with a cotton swab. Cells that had invaded the lower surface of the membrane were fixed, stained, and counted under a microscope.

### 4.7. Apoptosis Assay

Cell apoptosis was analyzed using an Annexin V-APC/7-AAD Apoptosis Detection Kit (Keygen Biotech, Nanjing, China; KGA1024) in both non-decidualized and decidualized HESCs. Non-decidualized cells were treated for 48 h with PCB118 (10^−10^–10^−8^ M) alone or combined with miRNA/plasmid, with PCB118 added during the final 24 h. Decidualized cells were processed as described in Section 4.1 and Section 4.3. After treatment, cells were stained with Annexin V-APC and 7-AAD in strict accordance with the manufacturer’s protocol and analyzed using a BD FACSCanto™ II Flow Cytometer (BD Biosciences, San Jose, CA, USA). Data acquisition and analysis were performed with BD FACSDiva™ Software (Version 8.0.1).

### 4.8. Molecular Biology Assays

#### 4.8.1. RNA and miRNA Extraction and qRT-PCR

Total RNA was isolated using Trizol reagent (Aidlab, Beijing, China; R401-01) according to the manufacturer’s instructions. RNA purity was assessed by 260/280 ratios (1.8–2.1) and agarose gel electrophoresis, indicating minimal protein contamination.

For PCR analysis of ILK and downstream signaling genes, cells were treated with PCB118, ILK plasmid, miR-542-3p mimic, or corresponding combinations, with appropriate controls included. cDNA synthesis was performed from 4.43 μg of total RNA after genomic DNA removal using the gDNA Eraser Premix (PrimeScript™ RT Reagent Kit, Takara, Kyoto, Japan; RR037A). Reverse transcription was carried out in a final volume of 20 μL, using a combination of oligo (dT) and random hexamer primers, at 37 °C for 15 min, followed by 85 °C for 5 s to inactivate the enzyme. Reactions were performed in technical triplicate, and cDNA was diluted 1:8 prior to qPCR.

Quantitative RT-PCR was performed using SYBR Premix Ex Taq (Takara, Kyoto, Japan, RR420A) on an ABI 7500 Real-Time PCR System (Applied Biosystems, Waltham, MA, USA). Each 20 µL reaction contained 0.4 µL of each 10 µM primer (listed in Table 1) and 2 µL of diluted cDNA. Cycling conditions were 95 °C for 3 min, followed by 40 cycles of 95 °C for 5 s and 60 °C for 30 s, melting from 58 °C to 95 °C. Melt curve analysis confirmed specific amplification, and no-template controls showed no amplification. Minus-RT controls were included for all mRNA assays to exclude genomic DNA contamination, particularly for primers spanning single-exon regions. 

Amplification efficiencies for all target and reference genes were determined using standard curves generated from five serial dilutions of pooled cDNA samples, each run in technical triplicate. Standard curves were generated by plotting Cq values against the logarithm of template concentration, and amplification efficiency (E) was calculated from the slope of the linear regression using the formula: E = 10^(−1/slope). The amplification efficiencies for all assays ranged from 94.7% to 103.4%, with correlation coefficients (R^2^)≥ 0.99 for the genes studied (Supplementary Table S1). Reference gene stability was assessed across all experimental conditions using the GeNorm algorithm. GAPDH exhibited the lowest M value (M < 0.5), indicating the highest expression stability among the candidate reference genes tested. Although the use of multiple reference genes and their geometric mean is generally recommended, the pairwise variation (V) analysis indicated that inclusion of additional reference genes did not significantly improve normalization stability (V < 0.15) and the high stability of GAPDH observed across all samples suggests that the impact on the present conclusions is likely minimal. Therefore, the use of a single reference gene (GAPDH) was considered appropriate in accordance with MIQE guidelines (Supplementary Tables S3 and S4, Figure S2).

Relative mRNA expression levels were calculated using the 2^−ΔΔCt^ method as amplification efficiency differences between target and reference genes were within an acceptable range (<10%). In parallel, efficiency-corrected calculations based on the Pfaffl method were performed as a validation step, and yielded highly consistent results (Supplementary Table S2). The use of a single reference gene was therefore considered appropriate and did not materially affect the interpretation of relative expression trends.

All qRT-PCR experiments included at least three independent biological replicates, each measured in technical triplicate. Technical replicates were averaged prior to statistical analysis. Variability is presented as mean ± SD of biological triplicates. Given the inherent variability of RT-qPCR measurements, particularly for small fold changes, results are interpreted as relative expression differences under defined experimental conditions rather than absolute quantitative changes.

For miRNA quantification, total RNA was isolated as described above, but an additional miRNA enrichment step was performed. cDNA was synthesized from 1.26 μg of total RNA using the miScript II RT Kit (Qiagen, Hilden, Germany; 218161) according to the manufacturer’s protocol. Reverse transcription reactions were performed in technical triplicate, and cDNA was diluted 1:3 prior to qPCR.

Quantitative RT-PCR for miRNA was carried out using the miScript SYBR Green PCR Kit (Qiagen, Hilden, Germany; 218073) with specific primers for miR-542-3p and U6 (internal control), as detailed in Table 1. Each 20 µL reaction contained 1 µL of each 10 µM primer and 2 µL of diluted cDNA. Cycling conditions were 95 °C for 15 min, followed by 40 cycles of 94 °C for 15 s, 55 °C for 30 s, and 70 °C for 30 s, melting from 65 °C to 95 °C. All reactions were performed in triplicate.

**Table 1:** Primers for RT-qPCR.

Genes	Primers (5′–3′)
miR-542-3P	5′-GTCGTATCCAGTGCAGGGTCCGAGGTATTCGCACTGGATACGACTTTCAGTT-3′
	5′-TGTGACAGATTGATAACTGAAA-3′
U6	5′-CGCTTCGGCAGCACATATAC-3′
	5′-AAATATGGAACGCTTCACGA-3′
ILK	5′-TGTGGAGTTTTGCAGTGCTT-3′
	5′-CGCTTTGCAGGGTCTTCATT-3′
TGF-β1	5′-CAGCAACAATTCCTGGCGATACCT-3′
	5′-CGCTAAGGCGAAAGCCCTCAAT-3′
Smad2	5′-GTCTCCAGGTATCCCATCG-3′
	5′-TTAGGATCTCGGTGTGTCGG-3′
VEGF	5′-GGCAGAAGGAGGAGGGATTT-3′
	5′-CCTGTCTGCTCTGGTATGATTGG-3′
MMP9	5′-CAGTCCACCCTTGTGCTCTTCCCTG-3′
	5′-ATCTCTGCCACCCGAGTGTAACCA-3′
GAPDH	5′-GCACCGTCAAGGCTGAGAAC-3′
	5′-TGGTGAAGACGCCAGTGGA-3′

#### 4.8.2. Western Blot Analysis

Proteins were extracted using RIPA lysis buffer (Beyotime, Shanghai, China), and concentrations were determined with a BCA Protein Assay Kit (Beyotime, Shanghai, China). Western blot analysis of ILK, TGF-β1, Smad2, VEGF, and MMP9 was performed using specific primary antibodies, with cells subjected to PCB118, ILK plasmid, miR-542-3p mimic, or combinations, and appropriate controls. Samples were separated on 10% SDS-PAGE gels, transferred to PVDF membranes (Millipore, Billerica, MA, USA), and probed with the following primary antibodies: anti-ILK (1:5000; Abcam, Cambridge, UK; ab52802), anti-TGF-β1 (1:200; Boster, Pleasanton, CA, USA; A0246), anti-Smad2 (1:1000; Affinity Biosciences, Cincinnati, OH, USA; AF6354), anti-VEGF (1:200; Boster, Pleasanton, CA, USA; A00902), anti-MMP9 (1:800; Abcam, Cambridge, UK; ab76003), and anti-GAPDH (1:1000; Abcam, Cambridge, UK; ab9485) and quantified using Quantity One software (version 4.6.8; Bio-Rad, Hercules, CA, USA).

#### 4.8.3. Tube Formation Assay

Decidualized HESCs in the logarithmic growth phase were seeded into 6-well plates (2 × 10^5^ cells/well) and divided into five groups: (1) decidualized control (PBS), (2) miR-542-3p mimics, (3) 10^−9^ M PCB118 alone, (4) miR-542-3p mimics plus 10^−9^ M PCB118, and (5) pcDNA3.1-ILK plasmid plus 10^−9^ M PCB118. Cells were induced to decidualize for 48 h, followed by 48 h of transfection as described in Section 4.3, with PCB118 (10^−9^ M) added during the final 24 h. After the 96 h total culture period, conditioned media were collected, centrifuged, and used to resuspend HUVECs. HUVECs were purchased from American Type Culture Collection (ATCC, Manassas, VA, USA; PCS-100-010). HUVECs were incubated in F12K medium (ATCC, Manassas, VA, USA) containing 10% FBS (fetal calf serum, Clark Bioscience, Melbourne, VIC, Australia), 1% ECGS (endothelial cell growth supplement, Sigma-Aldrich, St. Louis, MO, USA), and 0.1 mg/mL heparin (Sigma-Aldrich, St. Louis, MO, USA) in a humidified incubator containing 5% CO_2_ at 37 °C. Matrigel (BD Biosciences, San Jose, CA, USA) was thawed overnight, and 24-well plates were pre-cooled on ice. Each well was coated with 100 μL Matrigel, incubated at 37 °C for 30–60 min to allow gel formation. Decidualized cell culture supernatants were collected, centrifuged, and used to resuspend HUVECs, which were seeded onto Matrigel-coated wells (1 × 10^5^ cells/well) and incubated for 6 h at 37 °C with 5% CO_2_. Three images per group were captured at 100× magnification for vascular formation analysis.

### 4.9. Animal Experiments

Female CD-1 mice (3 weeks old) were housed under controlled conditions (14 h light/10 h dark cycle) and acclimatized for one week. A total of 24 mice were randomly assigned to two groups (n = 12 per group): a control group, which received sesame oil (vehicle, Sigma-Aldrich, St. Louis, MO, USA) via oral gavage for one month, and a PCB118 low-dose group, which received 10 µg/kg PCB118 (AccuStandard, New Haven, CT, USA; purity ≥ 99%) dissolved in sesame oil via oral gavage for one month. This dosing regimen is consistent with previously reported reproductive toxicity studies and estimated to represent the mean and even lower concentration (9.05–18 ng/g/lipid of PCB118) to which women are exposed [37,38]. Ovulation was induced by intraperitoneal injection of human menopausal gonadotropin (HMG, 10 IU, Sigma-Aldrich, St. Louis, MO, USA), followed 48 h later by human chorionic gonadotropin (HCG, 7.5 IU, Sigma-Aldrich, St. Louis, MO, USA). Subsequently, females were paired with untreated males in a 1:1 ratio for mating. The presence of a vaginal plug was designated as gestation day 0 (GD0). Out of 12 mice per group, 7 successfully became pregnant. Pregnant female mice were euthanized on GD8, and endometrial tissues were collected for subsequent analysis. All procedures were approved by the Institutional Ethics Committee of Zhongnan Hospital, Wuhan University (Approval No. 2021010) and performed following established ethical guidelines.

### 4.10. Statistical Analysis

All analyses were performed by SPSS version 26.0 and GraphPad Prism version 10.0. Data are presented as mean ± standard deviation (SD) from at least three independent experiments. Differences between the two groups were determined using Student’s *t*-test. For multiple comparisons, one-way analysis of variance (ANOVA) was used, followed by Tukey’s post hoc test. Differences were considered statistically significant at * *p* < 0.05, ** *p* < 0.01, and *** *p* < 0.001; ns indicates not significant.

## 5. Conclusions

In conclusion, this study provides evidence that PCB118 exposure is associated with impaired decidualization and angiogenesis, potentially involving dysregulation of the miR-542-3p/ILK signaling axis. This finding indicates that exposure to the persistent organic pollutant PCB118 may contribute to molecular alterations in decidual stromal cells and vascular endothelial cells, thereby contributing to defective endometrial remodeling during early pregnancy. These results expand the current understanding of how environmental pollutants interfere with key reproductive processes at the molecular level.

From a translational perspective, dysregulation of the miR-542-3p/ILK axis may serve as a potential molecular indicator for assessing PCB118-related reproductive risk. Moreover, therapeutic modulation of this pathway, such as inhibition of miR-542-3p or restoration of ILK activity, may represent a potential strategy to ameliorate decidualization and angiogenesis defects induced by environmental toxicants.

Overall, this work offers a mechanistic framework that is consistent with the involvement of microRNA-mediated regulation in PCB118-associated reproductive dysfunction, while further studies are required to establish direct causal relationships. These findings may contribute to a better understanding of environmentally associated reproductive disorders and support future studies aimed at protecting female reproductive health in the context of persistent environmental pollution.

## Data Availability

The original contributions presented in this study are included in the article. Further inquiries can be directed to the corresponding authors.

## Supplementary Materials

The following supporting information can be downloaded at: https://www.mdpi.com/article/10.3390/ijms27093771/s1.

## Abbreviations

The following abbreviations are used in this manuscript:

PCB118	2,3′,4,4′,5-Pentachlorobiphenyl
miRNAs	MicroRNAs
ILK	Integrin-linked kinase
HESCs	Human endometrial stromal cells
HUVECs	Human umbilical vein endothelial cells
PRL	Prolactin
IGFBP-1	Insulin-like growth factor binding protein-1
TGF-β1	Transforming growth factor beta 1
Smad2	SMAD family member 2
VEGF	Vascular endothelial growth factor
MMP9	Matrix metallopeptidase 9
EDCs	Environmental endocrine disruptors
PCBs	Polychlorinated biphenyls
cAMP	Cyclic Adenosine Monophosphate
MPA	Medroxyprogesterone 17-acetate
DMSO	Dimethyl Sulfoxide
qRT-PCR	Quantitative Reverse Transcription Polymerase Chain Reaction
ELISA	Enzyme-Linked Immunosorbent Assay
MTT	Methylthiazolyldiphenyl-tetrazolium bromide
UTR	Untranslated region
3′-UTRs	3′ Untranslated Regions
BMP7	Bone Morphogenetic Protein 7
pcDNA3.1-ILK	pcDNA3.1 vector containing Integrin-linked Kinase gene
DMEM/F12	Dulbecco’s Modified Eagle Medium/Nutrient Mixture F-12
Opti-MEM	Opti-Minimum Essential Medium
PBS	Phosphate-Buffered Saline
GD0	Gestation day 0
ANOVA	Analysis of Variance
SD	Standard Deviation
CDS	Coding sequence
dbcAMP	Dibutyryl cAMP
NC	Negative control
p-Smad2	Phosphorylated Smad2
