# Supplementary material for: PCB118 Is Associated with Impaired Decidualization and Angiogenesis Through miR-542-3p–Mediated Regulation of ILK Signaling
Source: Int J Mol Sci. 2026 Apr 23;27(9):3771. doi: 10.3390/ijms27093771 (PMC13163928; doi:10.3390/ijms27093771)
Supplement: Supplementary file 1 [file ijms-27-03771-s001.zip › ijms-4179761-supplementary.pdf]

Supplementary Materials

1. Amplification Efficiency and Pfaffl Correction

Amplification efficiencies for all target and reference genes ranged from 94.7% to 103.4%, falling within the acceptable range of 90–110%. As the efficiency differences between target and reference genes were less than 10%, relative expression levels were primarily calculated using the $2^{-\Delta\Delta Ct}$ method.

To further validate the robustness of the results, relative expression levels were additionally calculated using the Pfaffl method, which incorporates individual amplification efficiencies. The efficiency-corrected results were highly consistent with those obtained using the $2^{-\Delta\Delta Ct}$ method.

Supplementary Table S1. Amplification Efficiency and Linear Dynamic Range of All RT-qPCR Assays

Genename	Slope	R^2	Efficiency (%)	Linear Range (Cq)
GAPDH	-3.45	0.998	94.7	16.26–30.11
ILK	-3.28	0.992	101.2	20.18–33.79
TGFβ1	-3.44	0.993	95.5	20.11–33.86
Smad2	-3.34	0.991	99.2	21.86–31.56
VEGFa	-3.24	0.995	103.4	18.71–31.87
MMP9	-3.42	0.995	96.2	19.20–32.15
U6	-3.34	0.999	99.2	18.86-31.56

Supplementary Table S2. Comparison of relative expression levels calculated using the $2^{-\Delta\Delta Ct}$ method and the Pfaffl method. Fold changes were recalculated using the Pfaffl method incorporating individual amplification efficiencies for both target and reference genes. The efficiency-corrected values remained highly consistent with the original ΔΔCt-based results. Across all genes and experimental conditions, the percentage difference between the two methods was less than 10%, indicating strong concordance between the two calculation approaches.

Gene	Condition	$2^{-\Delta\Delta CT}$ Fold Change	Pfaffl Fold Change	% Difference
ILK	PCB	0.466	0.462	0.8
	mimic+ILK-plasmid	0.747	0.736	1.4
	mimic	0.401	0.412	2.7
	ILK-plasmid	1.312	1.288	1.8
	mimic+PCB	0.197	0.196	0.5
TGFβ1	PCB	0.553	0.538	2.7
	mimic+ILK-plasmid	0.791	0.725	8.3
	mimic	0.501	0.512	2.1
	mimic+PCB	0.248	0.256	3.2
VEGF	PCB	0.687	0.674	1.8
	mimic	0.636	0.632	0.6
	mimic+PCB	0.314	0.296	5.7
	ILK+PCB	0.967	0.95	1.7
MMP9	PCB	0.56	0.568	1.4
	mimic	0.489	0.499	2
	mimic+PCB	0.254	0.261	2.7
	ILK+PCB	1	0.996	0.4

2. Reference Gene Validation

Supplementary Table S3. Stability ranking of candidate reference genes based on geNorm analysis.GAPDH consistently ranked as the most stable reference gene across all experimental groups. TBP and ACTB showed comparable stability, with minor variations in ranking between conditions.

	rank	PCB	PCB+mimic	PCB+ILK	ILK
1	1	GAPDH	GAPDH	GAPDH	GAPDH
2	1	TBP	ACTB	ACTB	ACTB
3	3	ACTB	TBP	TBP	TBP

Supplementary Table S4. Average expression stability (M values) of candidate reference genes across experimental conditions.All candidate reference genes exhibited low M values (<0.5), indicating acceptable expression stability. Notably, M values were consistently below 0.2 across all conditions, suggesting high overall stability of the normalization system.

	**remaining_control_genes_Num**	PCB	PCB+mimic	ILK plasmid	mimic
1	3	0.112773402	0.032278509	0.084464311	0.107129404
2	2(GAPDH+ACTB)	0.095498076	0.02365857	0.053523673	0.033251988

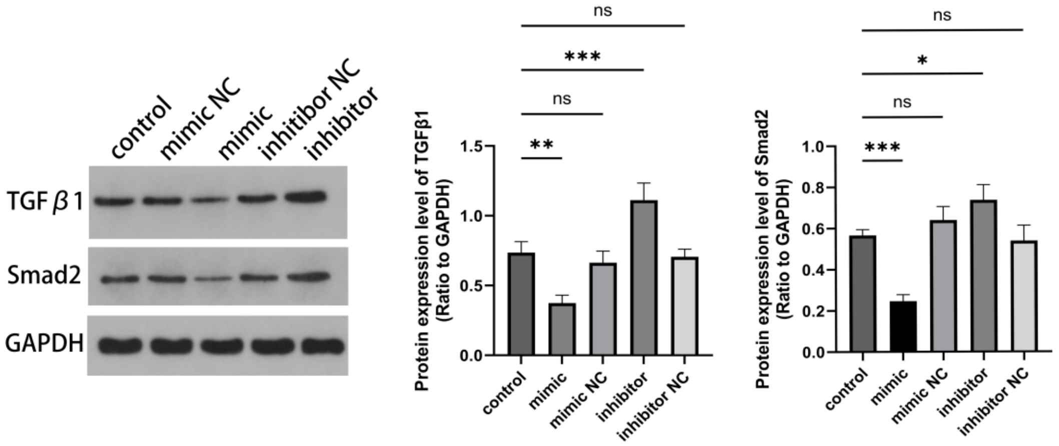

Supplementary Figure S1. Loss-of-function validation of miR-542-3p in decidualized HESCs. Decidualized HESCs were transfected with miR-542-3p inhibitor or inhibitor negative control (NC). Protein expression levels of TGF-β1 and Smad2 were assessed by Western blot. Quantitative analysis shows that inhibition of miR-542-3p significantly increased TGF-β1 and Smad2 expression compared with inhibitor NC. Data are presented as mean ± SD (n = 3). * p < 0.05, ** p < 0.01,***p < 0.001 versus control; ns, not significant.

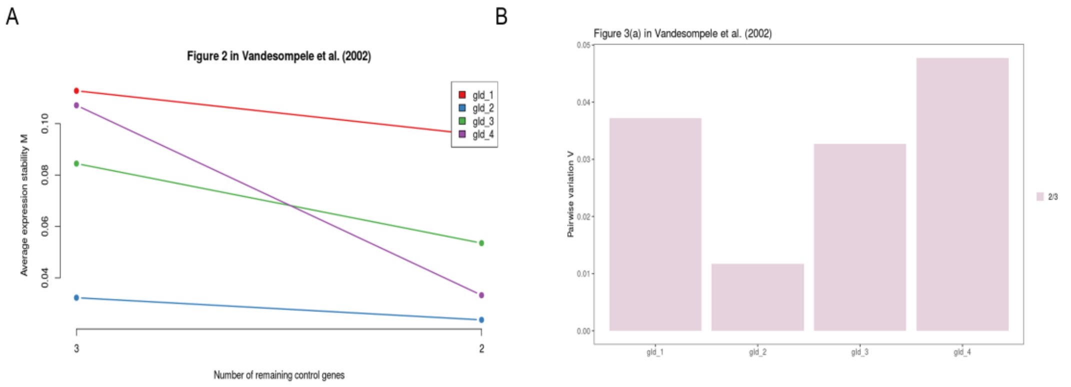

Supplementary Figure S2. geNorm analysis of candidate reference genes. (A) Average expression stability (M values) of GAPDH, ACTB, and TBP across all experimental conditions. Lower M values indicate higher stability. (B) Pairwise variation (Vn/n+1) analysis for determining the optimal number of reference genes. The pairwise variation value V2/3 was consistently below the recommended threshold of 0.15, indicating that the inclusion of additional reference genes does not significantly improve normalization accuracy.gld_1=PCB, gld_2=PCB+mimic, gld_3=ILK plasmid, gld_4=mimic
